# Association between body mass index and the risk of bacterial vaginosis: a systematic review and meta-analysis

**DOI:** 10.1097/MS9.0000000000003655

**Published:** 2025-07-25

**Authors:** Omar Khalid Samir Abdelkader, Noheir Ashraf Ibrahem Fathy Hassan, Amro Mahmoud Radi, Mennatallah Kamal, Karrar Hazim Abdul-Karim Al-Qizwini, Abdelrahman Ezzat, Helen A.O. Popoola-Samuel, Karim Aiash, Ayaan Arora, Amir Elissawy, Eman A. Toraih, Yusef Hazimeh, Hani Aiash

**Affiliations:** a Port Said University Faculty of Medicine, Port Said, Egypt; b Faculty of Medicine, Aswan University, Aswan, Egypt; c Obstetrics and Gynecology Department, New Ahmadi Hospital, Kuwait; d Suez Canal University, Ismailia, Egypt; e Al-Mustansiriyah University College of Medicine, Baghdad, Iraq; f College of Medicine, Royal College of Surgeons in Ireland, Dublin, Ireland; g St.George’s School of Medicine, Grenada, West Indies; h Jamesville Dewitt high school, Syracuse, New York, USA; i Christian Brothers Academy, Syracuse, New York, USA; j St. John’s University, Jamaica, New York, USA; k Surgery Department, Tulane University School of Medicine, New Orleans, USA; l Genetics Unit, Histology and Cell Biology Department, Suez Canal University School of Medicine, Ismailia, Egypt; m SUNY Upstate Medical University, Syracuse, New York, USA; n Department of Internal Medicine, Division of Endocrinology, American University of Beirut, Beirut, Lebanon

**Keywords:** bacterial vaginosis, body mass index, microbiota, overweight, risk factor, underweight

## Abstract

**Introduction:**

Bacterial vaginosis, resulting from vaginal microbiota imbalance and lactobacilli depletion, is the leading cause of abnormally appearing discharge in reproductive-aged women. Bacterial vaginosis is also associated with risk for sexually transmitted infections, preterm birth, and pelvic inflammatory disease. This meta-analysis assessed the association between the risk of bacterial vaginosis (BV) and body mass index (BMI).

**Methods:**

We systematically searched electronic databases using terms for weight, vaginosis, obesity, and BMI. We also reviewed gray literature, reference lists, and trials registries and sought the advice of experts. We calculated the overall odds ratios (OR) and the 95% confidence intervals (CI) using fixed-effect model for homogenous data and random-effects models for heterogenous data.

**Results:**

Eight observational studies (*n* = 22 190) were included, with quality assessment scores ranging from 7 to 9. Compared with normal-weight women (BMI 18.5–24.9), underweight women (BMI <18.5) had significantly higher prevalence of bacterial vaginosis (OR: 1.23; 95% CI: 1.12–1.36; *P* < 0.001). No significant associations were found for overweight (OR: 1.19; 95% CI: 0.95–1.48; *P* = 0.13) or obesity (OR: 1.29; 95% CI: 0.91–1.82; *P* = 0.15).

**Conclusion:**

Our findings suggest a significant association between underweight status and an increased likelihood of being diagnosed with bacterial vaginosis. However, causality cannot be established due to the observational nature of the included studies. Further prospective research is needed to confirm this relationship.

## Introduction

Bacterial vaginosis (BV) is a condition that occurs frequently in women within their reproductive years because their normal vaginal microbial balance becomes disrupted. This vaginal microbial imbalance occurs when *Lactobacillus* protective species diminish whereas anaerobic bacteria grow. BV exists alongside specific bacteria such as *Gardnerella vaginalis, Megasphaera* species, *Atopobium vaginae, Dialister* species, *Mobiluncus* species, *Sneathia amnii, Sneathia sanguinegens*, Porphyromonas species and Prevotella species^[1,2]^. BV affects about 29% of women across the United States but vary depending on different population groups and their geographic locations[3]. BV presents direct symptoms including irregular discharge and discomfort while leading to serious health complications. It also increases the chance of getting sexually transmitted infections and increases both the likelihood of preterm deliveries and pelvic inflammatory disease occurrences^[4–7]^. The potential complications associated with BV demonstrate the critical need to determine what factors contribute to its development and persistence.

**HIGHLIGHTS**

- First systematic review and meta-analysis assessing the relationship between BMI and bacterial vaginosis (BV).
- Underweight women (BMI < 18.5) had a significantly higher prevalence of BV compared to normal-weight individuals (OR: 1.23; 95% CI: 1.12–1.36; *P* < 0.001).
- No significant association was found between BV and overweight or obesity.
- Findings identifies key research gaps, urging further prospective studies on hormonal, immunological, and microbiota-related mechanisms linking BMI and BV.

Recent research attention has been drawn to body mass index (BMI) since new studies suggest that weight fluctuations might affect vaginal microbiota[8]. Several biological mechanisms could explain this relationship. Adipose tissue contributes to hormone regulation particularly for estrogen levels and this process affects vaginal pH and microbiota composition[9]. Additionally, The systemic inflammation linked to various BMI levels influences immune reactions and microbial equilibrium^[10,11]^.

The relationship between BV and BMI remains unclear since research findings about their connection show inconsistent results. Research suggests that women who have a higher BMI face greater BV risk because of hormonal imbalances together with inflammatory changes^[12–14]^. Conversely, some studies show that overweight and obese women tend to experience lower BV prevalence^[15,16]^, which may be due to differences in their vaginal microbiota populations or protective hormonal differences. Some research studies demonstrate no connection whatsoever between BMI measures and BV[17]. These contradictory research findings create difficulties for healthcare professionals who aim to identify vulnerable groups and implement specific screening programs.

To address this knowledge gap, we performed a systematic review and meta-analysis to explore in depth the connection between BMI and the risk of BV. Our study aims to evaluate if being underweight, overweight or obese affects BV risk differently compared to individuals with a normal BMI.

## Methods

This study was conducted and reported in accordance with the Preferred Reporting Items for Systematic Reviews and Meta-analysis (PRISMA) guidelines for reporting systematic reviews and meta-analyses[18]. The research protocol for this systematic review has been submitted to the International Prospective Register of Systematic Reviews (PROSPERO).

### Search strategy

We conducted a systematic review of the major electronic databases, PubMed/MEDLINE, SCOPUS and Web of Science, without language or date restriction. The search strategy employed (“body mass index” OR “BMI” OR “weight” OR “overweight” OR “underweight” OR “obesity”) and (“vaginosis” OR “vaginal dysbiosis” OR “BV”). In addition, we also manually searched reference lists of included studies, examined clinical trial registries, and interviewed experts in the field to help find any additional or unpublished information. Gray literature sources, including conference proceedings, dissertations, and reports by relevant organizations, were also pursued.

### Eligibility criteria

We included observational studies such as case-control, cohort studies along with cross-sectional studies and randomized controlled trials that investigated how body mass index (BMI in kg/m^2^) relates to bacterial vaginosis (BV). We included all studies published prior to 15 January 2025. The study population fell into four BMI categories: underweight (<18.5 kg/m^2^), normal weight (18.5–24.9 kg/m^2^), overweight (25.0–29.9 kg/m^2^), and obese (≥30 kg/m^2^). BV was either diagnosed using either the Nugent score or Amsel’s criteria. The Nugent score assess vaginal microbiota using Gram stain analysis, and classifies results as normal for scores 0–3, intermediate flora for scores 4–6, and positive BV for scores 7–10[19]. Amsel’s criteria diagnose BV based on four clinical signs: a homogeneous grayish-white discharge, a positive Whiff test, vaginal pH above 4.5, and the detection of clue cells on microscopy. The presence of three or more clinical signs from Amsel’s criteria leads to a BV diagnosis[20]. Women from the general population with normal BMI served as the primary comparator.

We excluded case reports or series with under 10 participants as well as studies lacking BMI group or BV risk group categorization. Non-original research, animal studies, conference abstracts without full text, and duplicate publications were also excluded.

### Screening

Two authors screened the retrieved studies independently according to the inclusion and exclusion criteria to ensure accuracy and minimize errors. First, they screened the titles and the abstracts to determine their relevance. Studies that met the inclusion criteria underwent full-text screening for final selection.

### Data extraction

Data extraction was performed using a standardized extraction sheet by two authors independently to ensure accuracy and consistency. Variables extracted for the baseline and summary included the first author’s last name, date, study design, country, sample size, age, BMI classification, and BV diagnostic method. The risk of BV according to the Nugent score or Amsel’s criteria was extracted as an outcome.

### Quality assessment

The risk of bias in the included studies was assessed using the Newcastle–Ottawa Scale for cohort studies[21], and a modified version for cross-sectional studies. Two reviewers independently assessed each study and resolved any disagreement by discussion or referral to a third reviewer.

### Statistical analysis

This meta-analysis has been performed using Review Manager Software (RevMan v5.4.1). The statistical representation of data included odds ratios (OR) paired with 95% confidence intervals (CIs). *P* <0.05 has been taken for significance. If heterogeneity wasn’t observed between the studies, the fixed effects model (Mantel–Haenszel method) was applied, while the random effects model (DerSimonian–Laird method) was applied when significant heterogeneity was observed. For heterogeneity, the Cochran’s Q test and the *I*^2^ test were used. If the value of the *P* was less than 0.05 or the value of the *I*^2^ was greater than 50%, heterogeneity was considered significant. The leave-one-out test was used to resolve the heterogeneity when found. Publication bias assessment (e.g., funnel plot, Egger’s test) and subgroup analysis was planned but was not performed as we had less than 10 studies.

## Results

### Literature search

**Figure 1.**
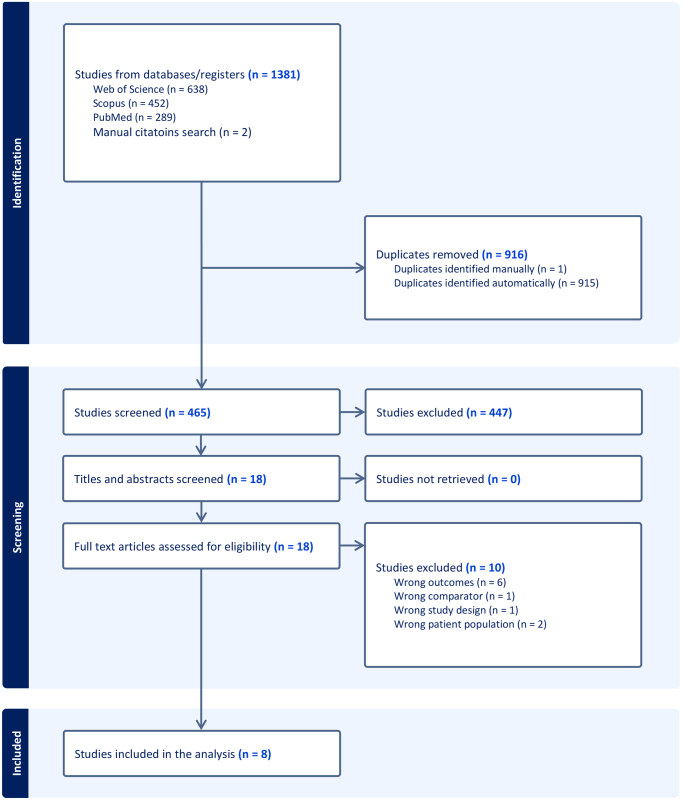
PRISMA flow diagram showing the study selection process.

A total of 1381 articles were identified from our search: 289 from PubMed, 452 from Scopus, 638 from WOS and 2 studies from manual citations search. After removing 916 duplicates, 465 articles remained for titles and abstracts screening. From the 465 articles we screened titles and abstracts for, we selected 18 articles for full-text review after excluding 447 articles because they failed to meet our inclusion criteria. After the full-text screening, 8 studies were included in our analysis^[6,12–17,22]^, as detailed in the PRISMA flow diagram (Fig. 1).

### Study characteristics

Eight observational studies, involving 22 190 participants were included in our study. Four of the included studies^[12–14,22]^ classified participants’ BV using the Nugent score. Two studies^[6,15]^ used Amsel’s criteria, while two studies used both the Nugent score and Amsel’s criteria^[16,17]^. Other study characteristics and baseline data are provided in Table 1.

**Table 1:** Baseline and summary of the included studies

Author, year [Reference]	Study design	Country	Sample size	BMI classification	BV diagnosis method	Age				
						Underweight (BMI < 18.5)	Normal (BMI 18.5–24.9)	Overweight (BMI 25–29.9)	Obese (BMI ≥ 30)	Overall
						Mean ± SD	Mean ± SD	Mean ± SD	Mean ± SD	Mean ± SD
Koumans, 2007^[22]^	Cross-sectional	USA	3,739	Underweight (BMI < 18.5) vs. Normal (BMI 18.5-24.9) vs. Overweight (BMI 25–29.9) vs. Obese (BMI ≥ 30).	Nugent score	N/A	N/A	N/A	N/A	N/A
Lokken, 2019^[16]^	Prospective cohort	Kenya	1946 (Total number of visits = 14319)	Underweight (BMI < 18.5) vs. Normal (BMI 18.5–24.9) vs. Overweight (BMI 25–29.9) vs. Obese (BMI ≥ 30).	Nugent score and Amsel’s criteria	28 ± 6.81	29.33 ± 6.67	31 ± 7.4	32 ± 5.9	30.33 ± 6.68
Brookheart, 2019^[12]^	Cross-sectional	USA	5918	Underweight (BMI < 18.5) vs. Normal (BMI 18.5–24.9) vs. Overweight (BMI 25–29.9) vs. Class I Obese (BMI 30–34.9) vs. Class II Obese (BMI ≥ 35).	Nugent score	23.2 ± 4.7	24.1 ± 5.4	25.6 ± 6	26.51 ± 6.16	25.3 ± 5.9
Bulut, 2020^[17]^	Cross-sectional	Turkey	106	Normal (BMI 18.5–24.9) vs. Overweight (BMI 25–29.9) vs. Obese (BMI ≥ 30).	Nugent score and Amsel’s criteria	N/A	N/A	N/A	N/A	32.1 ± N/A
Daubert, 2021^[15]^	Retrospective cohort	USA	4637 (Total number of visits = 56537)	Underweight (BMI < 18.5) vs. Normal (BMI 18.5–24.9) vs. Overweight (BMI 25–29.9) vs. Obese (BMI ≥ 30).	Amsel’s criteria	N/A	N/A	N/A	N/A	43 ± N/A
Zeng, 2023^[14]^	Cross-sectional	China	316	Underweight (BMI < 18.5) vs. Normal (BMI 18.5–24.9) vs. Overweight (BMI 25–29.9) vs. Obese (BMI ≥ 30).	Nugent score	N/A	N/A	N/A	N/A	N/A
Kalpana, 2024^[6]^	Cross-sectional	India	100	Underweight (BMI < 18.5) vs. Normal (BMI 18.5–24.9) vs. Overweight/Obese (BMI ≥ 25)	Amsel’s criteria	N/A	N/A	N/A	N/A	26.2 ± 3.5
Qi, 2024^[13]^	Cross-sectional	USA	5428	Underweight (BMI < 18.5) vs. Normal (BMI 18.5–24.9) vs. Overweight (BMI 25–29.9) vs. Obese (BMI ≥ 30).	Nugent score	N/A	N/A	N/A	N/A	32.4 ± 9.46

BMI: Body mass index, BV: Bacterial vaginosis, N/A: Not available, SD: Standard deviation, USA: United States of America.

The quality assessment scores of our included studies ranged from 7 to 9. The detailed quality assessment using the Newcastle–Ottawa scale is depicted in Table 2.

**Table 2:** Quality assessment of eligible studies

	Cohort studies								
	**Selection**				**Comparability**	**Outcome**			
Author (Year)	Representativeness of the exposed cohort	Selection ofthe non-exposed cohort	Ascertainment of exposure	Demonstration that the outcome of interest was not present at start of study	Comparability of cohorts on the basis of the design or analysis	Assessment of outcome	Was follow-up long enough for outcomes to occur	Adequacy of followup ofcohorts	Total score
Lokken (2019)	★	★	★	-	★	★	★	★	9
Daubert (2021)	★	★	★	-	★★	★	★	★	8
	**Cross-sectional studies**								
	**Selection**				**Comparability**	**Outcome**			**Quality**
**Author (Year)**	**Representativeness of the sample**	**Sample size**	**Non-respondents**	**Ascertainment of the exposure (risk factor)**	**Comparability of cohorts on the basis of the design or analysis**	**Assessment of outcome**	**Statistical test**		**Total Score**
Emilia (2007)	★	★	-	★★	★★	★★	★		9
Brookheart (2019)	★	★	-	★★	★★	★★	★		9
Bulut (2020)	★	★	★	★★	-	★★	★		8
Zeng (2023)	-	★	★	★	★	★★	★		7
Kalpana (2024)	-	★	★	★★	★★	★★	★		9
Qi (2024)	★	★	-	★★	★★	★★	★		9

★: One point, -: Zero points

### Underweight

**Figure 2.**
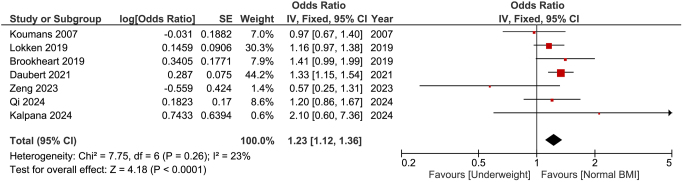
Forest plot comparing bacterial vaginosis in underweight women versus women with normal BMI. Data is presented as odds ratios (OR) with 95% confidence intervals (CI).

The pooled analysis revealed a significant increase in the prevalence of bacterial vaginosis among underweight participants compared to those with a normal BMI (OR: 1.23; 95% CI: 1.12–1.36; *P* < 0.001). We did not observe significant heterogeneity among the studies (*P* = 0.26, *I*^2^ = 21%) (Fig. 2).

### Overweight

**Figure 3.**
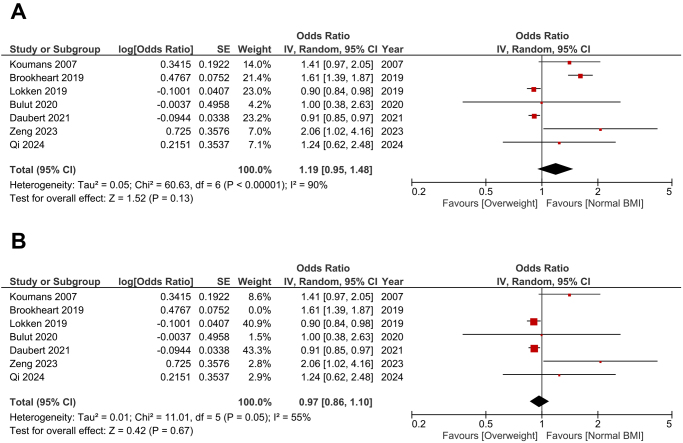
(A): Forest plot comparing bacterial vaginosis in overweight women versus women with normal BMI. (B): Leave-one-out test by removing the study by Brookheart et al (2019). Data is presented as odds ratios (OR) with 95% confidence intervals (CI).

The pooled analysis revealed no statistically significant difference in bacterial vaginosis prevalence between overweight participants and those with a normal BMI (OR: 1.19; 95% CI: 0.95–1.48; *P* = 0.13) (Fig. 3A). However, significant heterogeneity was found among the studies (*P* < 0.001, *I*^2^ = 93%). After the leave-one-out test was done by removing the study by Brookheart et al[12]. The heterogeneity decreased (*P* = 0.05, *I*^2^ = 55%), and the association remained insignificant (OR: 0.97; 95% CI: 0.86–1.10; *P* = 0.67) (Fig. 3B).

### Obesity

**Figure 4.**
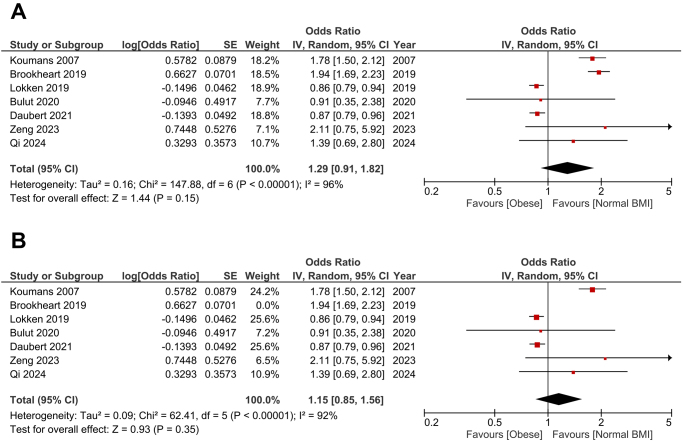
Forest plot comparing bacterial vaginosis in obese women versus women with normal BMI. (B): Leave-one-out test by removing the study by Brookheart et al (2019). Data is presented as odds ratios (OR) with 95% confidence intervals (CI).

The pooled analysis revealed no statistically significant difference in bacterial vaginosis prevalence between obese participants and those with a normal BMI (OR: 1.29; 95% CI: 0.91–1.82; *P* = 0.15) (Fig. 4A). However, significant heterogeneity was found among the studies (*P* < 0.001, *I*^2^ = 96%). After the leave-one-out test was done by removing the study by Brookheart et al[12], the heterogeneity wasn’t resolved (*P* < 0.001, *I*^2^ = 92%), and the association remained insignificant (OR: 1.15; 95% CI: 0.85–1.56; *P* = 0.35) (Fig. 4B).

## Discussion

This is the first meta-analysis assessing the association of body weight among women to the risk of developing bacterial vaginosis (BV). Our study detected that being underweight is significantly associated with more likelihood of being diagnosed with BV, while being overweight or obese is not associated with BV. However, causality cannot be established due to the cross-sectional nature of the data.

There is little research evaluating the relationship between BV and BMI. According to the findings of a prior large prospective cohort research that regularly measured BMI and disrupted the vaginal microbiota, obese women may be less likely to get BV. In a study by Lokken et al, obese Kenyan women had a roughly 20% decreased risk of BV, and a 10% decrease in BV among overweight women which was marginally significant, indicating a dose-response impact[16]. The same study reported higher rates of BV among underweight women with prevalence of 41% compared to 37.5% among lean, 35.2% among overweight, and 34.1% among obese women coming in agreement with our findings. A short study of copper IUDs in Thai women with a median age of 39 years revealed that BV was more common among those with a BMI <20 kg/m^2^, according to Kancheva et al, premenopausal women with a BMI of less than 18.5 also had a considerably higher risk of BV in this sample, albeit this difference was not statistically significant in adjusted analysis[23]. These findings come in contrast to other previous research which reported that the higher the BMI, the higher the prevalence of BV^[24–26]^. Teenagers in the United States who self-reported being overweight or obese before becoming pregnant had a higher chance of being diagnosed with BV during pregnancy than those who were underweight or normal before becoming pregnant (aOR 1.16, 95% CI 1.04–1.30)[26]. In a study by Lennard et al, 16–22 year-old South African women with higher BMI and HIV seronegative status had a greater liklehood of non-Lactobacillys-dominant vaginal microbiome (aOR 1.2, 95% CI 1.0–1.3)[24]. Researchers examined the association between 106 women of reproductive age and BV in a retrospective analysis. They found that there was no meaningful correlation between BV and BMI[17]. Another retrospective investigation by Daubert et al demonstrated that HIV-positive women with obesity (BMI >30 kg/m^2^) experienced lower BV rates compared to the reference group of women with normal BMI (18.5–24.9 kg/m^2^)[15]. When comparing obese women to non-obese participants, Raglan et al found that the former had a higher prevalence of high-diversity vaginal microbiota (depleted in *Lactobacillus* spp. and *Gardnerella* spp.) and a lower prevalence of Lactobacilli-dominated vaginal microbiota (*P* < 0.001)[8]. Several molecular factors could explain the observed 23% elevated BV occurrence among underweight women. Underweight status commonly involves nutritional deficits which then disrupt hormone regulation along with immune system operations. Women with low body weight have diminished estrogen levels which alter the environment necessary for healthy Lactobacillus colonization and thus threaten the stability of the vaginal epithelium[27]. Furthermore, immunological responses might be compromised by starvation, which may have an impact on the body’s capacity to preserve a healthy vaginal microbiota. An increased incidence of BV and a decrease in Lactobacilli are consistently linked to menstruation^[28,29]^. Ovulatory dysfunction is a condition that some obese and overweight women suffer[9], which may reduce their risk of incident BV. Furthermore, vaginal epithelial cells may contain more glycogen due to increased estrogen levels generated by adipose tissue[9]. A more ideal vaginal environment may be supported by increased glycogen, which encourages Lactobacillus colonization and lactic acid production[27]. Changes in gut flora brought on by nutrition or obesity may potentially account for a correlation between BMI and BV. According to an increasing amount of research, obesity is linked to a less varied gut microbiota, which is linked to several chronic illnesses[30]. Through bacterial translocation or similar host-immune traits, the vaginal microbiota may share changes with the gut microbiota[31]. Emerging evidence suggests that BMI affects not only the vaginal microbiome but also the skin and mucosal microbiomes, with systemic effects on immunity[32]. In obesity, the skin barrier function is usually impaired. It is characterized by increased pH, transepidermal water loss (TEWL), and altered lipid composition which can promote colonization by organisms such as Corynebacterium, Streptococcus, and Candida^[33–35]^. Meanwhile, underweight individuals may show different skin microbial patterns, possibly linked to immune alterations. When an individual is obese, dermal adipocytes become dysfunctional, resulting in reduced production of antimicrobial peptides (AMPs) like cathelicidin. This weakening of the skin’s innate immune defenses can allow the proliferation of pathogens such as *Staphylococcus aureus*^[36,37]^. This obesity-induced immunity disturbance impairs wound healing and increases susceptibility to skin infections[38]. Evidence of crosstalk between mucosal sites, such as the gut and skin, further supports the idea of systemic interactions influencing vaginal health^[39,40]^. Both low and high BMI appear to affect the microbiome and immune system in ways that could explain the link to BV. This link needs more in-depth prospective research to be well understood.

### Strengths and limitations

There are several strengths to this meta-analysis. With a sizeable sample size and a range of research populations, it is the first thorough synthesis of data pertaining to BMI and BV risk. These findings appear to be robust across several study contexts, as indicated by the minimal heterogeneity in the underweight analysis. Our thorough process, which includes sensitivity analyses and quality evaluation, also improves the dependability of our results.

However, there are a few limitations to consider. Our capacity to demonstrate causal links was limited by the cross-sectional nature of most of the included studies. Although no heterogeneity was observed in the analysis for underweight BMI, and we were able to reduce heterogeneity for overweight BMI through a leave-one-out test, substantial heterogeneity persisted in the analysis for obesity BMI. This residual heterogeneity may be attributed to several factors, including variations in the definitions and categorization of BMI across the included studies, as well as differences in the diagnostic criteria used for bacterial vaginosis, as some studies used Amsel’s criteria, while others used the Nugent score. Geographic variation may also have played a significant role; although it enhances the generalizability of our findings, the inclusion of studies from diverse countries such as the United States, China, Turkey, India, and Kenya could have introduced variability in populations, healthcare settings, and lifestyle factors that influence the observed outcomes.

Furthermore, due to inconsistent reporting across the included studies, we were unable to account for several potential confounding factors such as concurrent illnesses, hygiene practices including vaginal douching, sexual behavior, and antibiotic use. So, there is a possibility of residual confounding that was not eliminated. Additionally, the cross-sectional nature of the data limits our ability to assess longitudinal changes in BMI and their impact on BV risk over time. Future studies with more comprehensive data collection and prospective designs are needed to better understand and confirm these relationships. Additionally, the included studies did not report specific bacterial profiles associated with BV stratified by BMI categories. Therefore, we were unable to determine whether the types of bacteria contributing to BV differed between underweight and obese participants, limiting our ability to assess whether the microbiological patterns differed between different BMI categories.

More comprehensive studies of the pathways from underweight status to BV can help to develop targeted therapies. Research examining the relationship between diet, immune system functioning, and hormonal activity would provide valuable insights into this connection. Our analysis variations may be clarified through investigations that examine modifiers like dietary patterns, geographic placement and ethnic distinctions.

Moreover, the assessment of publication bias was limited by the low number of included studies (*n* = 8). Funnel plots and Egger’s test are statistical methods that are widely used to assess the presence of publication bias. However, their reliability is diminished when the number of analyzed studies is fewer than 10. Therefore, the existence of publication bias cannot be excluded, which may impact the results. If publication bias is present, it could lead to an overestimation of the association between BMI and bacterial vaginosis, particularly if studies with null or negative findings were less likely to be published.

Our findings demonstrate an association between underweight status and an increased likelihood of being diagnosed with bacterial vaginosis, highlighting a potential area for further research and public health attention. While this suggests that undernutrition may play a role in BV risk, it is important to note that causality cannot be established due to the observational nature of most of the studies included. Therefore, healthcare professionals may consider monitoring BMI in populations with high rates of undernutrition as part of a broader risk assessment strategy, while recognizing the need for more prospective studies to confirm this association.

BMI does not appear to be a reliable marker for assessing BV risk among overweight or obese individuals, as no significant association was observed. A comprehensive approach to BV diagnosis and prevention should continue to incorporate established risk factors including sexual activity patterns and vaginal douching, and hormonal contraceptive use. The findings of our study may be of interest not only to gynecologists, but also to dermatologists and experts from other specialties examining the impact of systemic influences like nutrition on the skin and mucosal microbiome.

## Data Availability

Data needed is available within the manuscript. Raw data will be shared upon request.

## Research registration unique identifying number (UIN)

Registry used: PROSPERO. Unique Identifying number or registration ID: CRD420250652467. Link: https://www.crd.york.ac.uk/PROSPERO/view/CRD42025065246.
